# Hypertension Associated With Hearing Loss and Tinnitus Among Hypertensive Adults at a Tertiary Hospital in South Africa

**DOI:** 10.3389/fneur.2022.857600

**Published:** 2022-03-16

**Authors:** Hlologelo Ramatsoma, Sean Mark Patrick

**Affiliations:** School of Health Systems and Public Health, Faculty of Health Sciences, University of Pretoria, Pretoria, South Africa

**Keywords:** auditory deficits, cardiovascular diseases, hypertension, hearing loss, tinnitus

## Abstract

**Introduction:**

Hypertension is one of the leading causes of morbidity and mortality worldwide, and has been associated with target organ damage. Effects of hypertension on the auditory system are varied and requires further investigation. This study aimed to investigate the association between hypertension and auditory deficits (hearing loss and tinnitus).

**Methods:**

This study employed a cross-sectional study including 106 (54.7% female) hypertensive adults aged 18–55 years, and 92 (52.2% female) non-hypertensive sex- and age-matched adults residing in South Africa. A data extraction sheet was used to obtain hypertension information from participants' medical files, and to subjectively obtain tinnitus status and characteristics among participants. Participants' hearing sensitivity—including extended high frequencies (EHF)—were measured using a diagnostic audiometer. The χ2 test determined the difference in auditory deficit prevalence between the study groups. Logistic regression was used to identify predictor variables associated with auditory deficits in the hypertensive group.

**Results:**

A hearing loss prevalence of 37.4% among hypertensive adults compared to 14.1% among the non-hypertensive group (*P* = 0.000, χ2 = 14.00) was found. The EHF pure-tone average among the hypertensive group was 44.1 ± 19.2 dB HL, and 20.0 ± 18.3 dB HL among the control group. Bilateral mild sensorineural hearing loss was the most common type of hearing loss among hypertensive adults. A higher prevalence of tinnitus (41.5%) was found in the hypertensive group compared to the control group (22.8%) (*P* = 0.008, χ2 = 7.09). In this study, 30.3% of hypertensive adults had tinnitus without hearing loss compared to 17.7% non-hypertensive adults. Factors associated with hearing loss included being between 50 and 55 years [adjusted Odds Ratio (AOR) = 3.35; 95% Confidence Interval (CI): 1.32–8.50; *P* = 0.011], having grade 2 hypertension (AOR = 4.18; 95% CI: 1.02–17.10; *P* = 0.048), and being on antihypertensive medication (AOR = 3.18; 95% CI: 1.02–9.87; *P* = 0.045). Tinnitus was associated with grade 3 hypertension (AOR = 3.90; 95% CI: 1.12–12.64; *P* = 0.033).

**Conclusions:**

Our study showed that hypertensive adults had a higher proportion of hearing loss and tinnitus compared to non-hypertensive adults. Findings suggest an association between hypertension and auditory deficits, demonstrating a need for integration of hearing healthcare services for hypertension management.

## Introduction

Hypertension, or high blood pressure (HBP) in the arterial walls, is an issue of global public health concern; and responsible for 8.5 million deaths due to ischaemic heart disease, stroke, renal diseases, and other vascular diseases globally (1, 2). Hypertension is defined as having a systolic blood pressure (SBP) of 140 millimeter of mercury (mmHg) and/or a diastolic blood pressure (DBP) of 90 mmHg (3, 4). Although the exact global prevalence of hypertension is unknown; the worldwide prevalence of hypertension is rising. In the year 2005, it was estimated that 26% (972 million people) of the world's population had hypertension (5). In 2010, 31.1% of the global adult population (1.38 billion people) had hypertension—marking a 5.1% increase in hypertension prevalence in 5 years (6). Low- and middle-income countries (LMICs), such as those in Sub-Saharan Africa, have been reported to have higher proportions of hypertensive cases compared to high-income countries (HICs), with a prevalence of 31.5 and 28.5%, respectively (6). Although hypertension prevalence of up to 60% have been reported in South Africa (7), the World Health Organization (WHO) estimates that 27.4 and 26.1% of men and women in South Africa are hypertensive, respectively (8).

Chronic exposure to HBP has been reported to lead to target organ damage (TOD) such as kidneys, brain, heart, and eyes (9–11). As a result, routine assessments of signs of hypertensive TOD have been recommended by the American College of Cardiology Foundation/American Heart Association Task Force on Practice Guidelines (ACCF/AHA) (12). The auditory system is not part of the hypertension TOD list to be routinely assessed as outlined by the ACCF/AHA, despite increasing evidence demonstrating an association between hypertension and auditory deficits (13–16). In this paper, auditory deficits are defined as having hearing loss and or tinnitus. Several mechanisms of action have been proposed explaining the effects of hypertension on the auditory system, such as HBP leading to increased blood viscosity which leads to increased resistance to blood flow and deprivation of oxygen to the body, organs, and tissue (17). Another mechanism is that HBP in the systemic arteries causes hemorrhage within the cochlea, thus compromising its structure and leading to hearing loss (18).

The high prevalence of hypertension globally and its reported association with auditory deficits deems it of public health importance. There is still much debate on the association between hypertension and hearing loss. Some authors have reported a higher prevalence of hearing loss among hypertensives while others observed similar hearing sensitivities between hypertensive and non-hypertensive participants. Recently, two studies in Nigeria reported a hearing loss prevalence of 38.5 and 12.83% among hypertensives (16, 19). Another study in India reported a sensorineural hearing loss (SNHL) prevalence of 36.7% among hypertensive adults—with a notable worsening of hearing sensitivity the higher the SBP and DBP (13). Contrary to this, two independent studies in Brazil and the United States of America (USA) reported comparable hearing thresholds between the hypertensive group and the non-hypertensive group (20, 21). The lack of association between hypertension and hearing loss in both studies could be attributed to age-related hearing loss confounding the results, as both studies that did not report an association used older adults as study participants.

There is a plethora of literature demonstrating the association between hypertension and tinnitus (22). One study in Brazil has reported a tinnitus prevalence of 43.7% among hypertensive adults (23). Interestingly, the 43.7% of the hypertensive participants who reported tinnitus in the study also had some degree of hearing loss. This is not surprising as reports suggest that 85–96% of individuals with hearing loss have tinnitus (24). Thus, it is difficult to attribute tinnitus to either hypertension or hearing loss in their study. It is important to determine the proportion of hypertensive adults with tinnitus but without hearing loss. Tinnitus may be a debilitating condition in some patients. Severe tinnitus has been reported to affect hearing, concentration, and sleep (25). Although some patients affected with tinnitus adjust without difficulty, others are severely affected by tinnitus leading to a reduced quality of life (25). In rare but probable cases, tinnitus leads to suicide (26).

Globally, the WHO estimates that 1.5 billion people or 20% of the population is affected by hearing loss; the majority of the people affected by hearing loss (1.16 billion) have mild hearing loss (27). The African region exclusively contributes 9% to that statistic. Simultaneously, the region is burdened with a high patient to clinician ratio resulting in significantly limited access to hearing healthcare services (28). The prevalence of hearing loss is estimated to increase to 2.5 billion by 2050 (27). This increase is mainly driven by persistent and growing risk factors; thus, it is crucial that factors associated with hearing loss are determined to facilitate prevention and or early management of hearing loss. There is extensive research chronicling the range of negative impacts that untreated hearing deficits have on an individual's life (29). Using the EuroQol-5 Dimension (EQ-5D) tool to assess the effects of various conditions such as hypertension, diabetes, blindness and depression, it was noted that hearing impairments yielded the biggest decrease in self-reported EQ-5D health-related quality of life (HRQoL) utility scores (30). Hearing loss has a negative impact on individuals' communication, cognitive, emotional and social functions, as well as their ability to obtain or retain employment thus resulting in increased economic burden. Therefore, the potential increase in hearing loss amongst hypertensive adults poses a threat to the already strained public health system resources, the economy, and individuals HRQoL.

The association between hypertension and auditory deficits has been an important subject of public health relevance globally. The prevention of auditory deficits is important as treatment is often difficult and costly. In 2019, the staggering global cost of hearing loss was estimated to be more than $981 billion (31). In South Africa, no research dedicated to the investigation of the association between hypertension and auditory deficits has been identified. This study sought to determine the association between hypertension and auditory deficits by determining the prevalence and characteristics of auditory deficits among hypertensive adults (compared to non-hypertensive adults) attending the out-patient clinic of Helen Joseph tertiary hospital, Johannesburg, South Africa. Furthermore, this paper systematically documents the association in order to influence public healthcare policy and clinical practice on the management of hypertensive adults in South Africa.

## Materials and Methods

### Study Design and Sample Size Determination

This study employed a quantitative cross-sectional study design in order to determine the prevalence and characteristics of hearing loss and tinnitus in hypertensive adults in South Africa. The sample size estimation for this study was based on the 38.5% hearing loss prevalence among hypertensives and 13.5% hearing loss prevalence among non-hypertensives reported in a similar study conducted in Nigeria (16). Using the sample size formula for two proportions described in Kelsey (32);


$$\begin{array}{l} n=\left(\frac{r+1}{r}\right)\frac{(\bar{p})(1-\bar{p}){\left(Z_{\beta }+Z_{\alpha /2}\right)}^{2}}{{(P_{1}-P_{2}\text{)}}^{2}}\\ n=\left(\frac{1+1}{1}\right)\frac{(0.26)(1-0.26){\left(0.84+1.96\right)}^{2}}{{\left(0.135-0.385\right)}^{2}} \end{array}$$

The minimum required sample size for a two-group design (hypertensive and non-hypertensive group) with α = 0.05 and 1–β = 0.80 was estimated to be 48 in each arm (a total of 96 participants). Previous similar studies have had sample sizes between 50 and 100 participants (13, 16, 20). The current study included 106 (54.7% female) hypertensive adults and 92 (52.2% female) sex-matched and age-matched non-hypertensive adults attending the out-patient clinic at the Helen Joseph hospital between September 2021 and October 2021.

The *inclusion criteria* for the experimental group were being an adult between ages 18 and 55 years of age, having been diagnosed with hypertension by a clinician and attending the out-patient polyclinic or the medical out-patient-department at Helen Joseph hospital. For the control group, the inclusion criteria were being non-hypertensive and between the ages 18 and 55 years attending the out-patient polyclinic or the medical out-patient-department at Helen Joseph hospital. The *exclusion criteria* for both groups included having an air-bone gap of ≥10 dB HL indicating a middle ear condition, co-morbidities such as HIV/ AIDS and diabetes mellitus known to be risk factors for hearing loss, history of hearing aid use, history of ear surgery performed on the participant, history of occupational noise exposure, history of ototoxic drugs use, history of cigarette smoking, history of stroke or head injury, and family history of hearing loss.

This study was granted ethical approval from the University of Pretoria Health Sciences Research Ethics committee (Ethics Reference No: 287/2021). All the study methods were in compliance with relevant ethical and clinical guidelines and regulations. Prior to participation, all participants signed an informed consent.

### Demographic Information and Blood Pressure Measurement

Demographic information obtained from the participants were sex and age. A data extraction sheet was created to obtain hypertension information from the study hypertension group's medical files. In this study, individuals were regarded as hypertensive if their medical records indicated a clinician-diagnosed hypertension. The items included in the data extraction sheet were, (I) the duration of hypertension diagnosis, (II) last recorded SBP and DBP, and (III) if the participant is on hypertensive medication. Each hypertensive participant's last recorded SBP and DBP were classified as per the WHO/International Society of Hypertension (ISH) grades of hypertension, which were: grade 1 (SPB = 140–159 and/or DBP = 90–99), grade 2 (SPB = 160–179 and/or DBP = 100–109), and grade 3 (SPB = ≥180 and/or DBP = ≥110) (33). In addition, participants with SPB = <140 and/or DBP = <90 were classified as controlled.

### Audiological Measurements

Both the study and control participants' auditory status were evaluated and reported. In order to determine the proportion of participants with tinnitus and the tinnitus characteristics in each group, a data sheet was developed with the following items; tinnitus presence, tinnitus laterality, and tinnitus sound (i.e., buzzing, hissing or pulsating). A qualified clinical audiologist performed otoscopic examination and audiometric evaluation on both groups. Both participants' ears were examined during otoscopy, and any impacted cerumen was removed prior to pure tone audiometry.

Hearing thresholds were measured using the Modified Hughson-Westlake threshold seeking method at octave frequencies 250–8,000 Hz, and extended high frequencies (EHF) 9,000, 10,000, 11,200, 12,500, 14,000, and 16,000 Hz for air conduction, and 500–4,000 Hz for bone conduction. The audiometer used in this study (KUDUwave Pro, eMoyo, South Africa) was a calibrated diagnostic audiometer (International Electrotechnical Commission 60645-1, 2017) with the capability to conduct diagnostic audiometry outside of a sound-treated booth (34). Nonetheless, audiometric measurements were conducted in a quiet room situated in the hospital's audiology department. A four-frequency pure-tone average (PTA) of audiometric thresholds at 500, 1,000, 2,000, and 4,000 Hz (PTA_0.5, 1, 2, 4_) was determined and used to classify the degree of hearing loss. The classification of the degree of hearing loss was arranged in accordance to the recently revised World Health Organization (WHO) hearing loss classification system as: normal hearing [ <20 decibel hearing level (dB HL)], mild hearing loss (20 to <35 dB HL), moderate hearing loss (35 to <50 dB HL), moderately severe hearing loss (50 to <65 dB HL), severe hearing loss (65 to <80 dB HL), profound hearing loss (80 to <95 dB HL), complete or total hearing loss/deafness (≥95 dB HL) (27). All participants with hearing loss as per the WHO criteria were included in the final calculation of the prevalence of hearing loss—regardless of the laterality of the hearing loss. In addition to this, the pure tone average for the EHFs (PTA_9,10,11.2,12.5,14,16_) was determined between the two study groups.

### Statistical Analysis

All statistical analysis were done using the Stata version 16.0 (StataCorp LLC, College Station, TX, USA). Descriptive statistics were used to summarize and describe participants' demographic and hypertension data such as sex, age, duration of hypertension diagnosis, and grade of hypertension. Frequency tables were used to report the distribution of auditory deficits in both study groups. The difference between the prevalence of auditory deficits in both groups were determined using the χ2 test for categorical variables; *P*-values of < 0.05 were used to determine level of significance. A logistic regression model was used to determine the risk factors for auditory deficits among hypertensive adults with various hypertensive characteristics. All hypothesis tests were done using a significance level of 0.05, Odds ratios and adjusted Odds ratios (OR; AORs) and their 95% confidence intervals were tabulated.

## Results

### Demographic Characteristics of Study Participants

A total of 198 participants were included in this study. One hundred and six participants were hypertensives while 92 were non-hypertensive controls. Just over half (54.7 and 52.2%) of the participants were females in both the hypertensive and non-hypertensive controls, respectively. The mean ages of the hypertensive and control participants were 45.1 ± 9.3 years and 43.3 ± 8.7 years, respectively. There was no statistically significant difference between the sex and age of both the groups (Table 1).

**Table 1:** Demographic characteristics of study participants.

	**Hypertensive *n* (%)**	**Non-hypertensive *n* (%)**	***P*-value (based on independent *t* test)**
Sample size	106	92	
**Sex**			0.72
Female	58 (54.7)	48 (52.2)	
Male	48 (45.3)	44 (47.8)	
**Age in years**			
18–29 years	8 (7.6)	7 (7.6)	
30–39 years	22 (20.6)	27 (29.4)	
40–49 years	32 (30.2)	29 (31.5)	
50–55 years	44 (41.5)	29 (31.5)	
Age (Mean ± SD)	45.1 (±9.3)	43.3 (±8.7)	0.17

### Hypertension Characteristics of Study Hypertensive Group

Table 2 shows the hypertension characteristics among the hypertensive participants. The majority of the hypertensive participants (48.1%) had controlled high blood pressure (Table 2). About 44.3% of the hypertensive participants had hypertension for the duration of 0–5 years, with the least number of participants (25.5%) having had hypertension for the duration of 6–10 years. Around 73.6% of the hypertensive participants were on antihypertensive medication.

**Table 2:** Hypertension characteristics among study hypertensives.

	**Hypertensives**
**Grade of hypertension**	***n*** **(%)**
Controlled (SPB <140/DBP <90)	51 (48.1)
Grade 1 (SPB ≥ 140–159/DBP ≥ 90–99)	30 (28.3)
Grade 2 (SPB ≥ 160–179/DBP ≥ 100–109)	12 (11.3)
Grade 3 (SPB ≥ 180/DBP ≥ 110)	13 (12.3)
**Duration of hypertension**	***n*** **(%)**
0–5 years	47 (44.3)
6–10 years	27 (25.5)
>10 years	32 (30.2)
**On antihypertensive medication**	***n*** **(%)**
Yes	78 (73.6)
No	28 (26.4)

### Prevalence and Characteristics of Auditory Deficits Among Participants

All participants in this study either had normal hearing or sensorineural hearing loss (SNHL). Participants with hearing loss with a conductive component (air-bone gap ≥10 dB HL) were excluded from this study as per the exclusion criteria. The prevalence of hearing loss was 37.4% in the hypertensive group and 14.1% in the non-hypertensive control group (Table 3).

**Table 3:** Prevalence and characteristics of auditory deficits among hypertensive and non-hypertensive adults.

	**Hypertensives n (%)**	**Non-hypertensives n (%)**
Sample size	106	92
Participants with hearing loss	40 (37.4)	13 (14.1)
**Hearing loss laterality**	***n*** **(%)**	***n*** **(%)**
Unilateral	16 (40.0)	6 (46.1)
Bilateral	24 (60.0)	7 (53.9)
Number of ears tested	212	184
**Degree of hearing loss (PTA**_**0.5, 1, 2, 4**_ **)—ear specific**	***n*** **(%)**	***n*** **(%)**
Normal	148 (69.8)	164 (77.4)
Mild	42 (19.8)	16 (7.5)
Moderate	13 (6.1)	3 (1.4)
Moderately severe	6 (2.8)	1 (0.5)
Severe	2 (0.9)	–
Profound	1 (0.5)	–
**EHF PTA**_**9,10,11.2,12.5,14,16**_ **–in both ears**	**(Mean** **±SD)**	**(Mean** **±SD]**
	44.1 ± 19.2	20.0 ± 18.3
**Participants with tinnitus**	***n*** **(%)**	***n*** **(%)**
	44 (41.5)	21 (22.8)
**Tinnitus laterality**	***n*** **(%)**	***n*** **(%)**
Unilateral	20 (45.4)	11 (52.4)
Bilateral	24 (54.6)	10 (47.6)
**Tinnitus sound**	***n*** **(%)**	***n*** **(%)**
Whooshing	5 (11.4)	4 (19.1)
Pulsating	6 (13.6)	1 (4.8)
Buzzing	13 (29.5)	3 (14.3)
Hissing	19 (43.2)	7 (33.3)
Screeching	1 (2.3)	6 (28.6)
**Participants with tinnitus without hearing loss**	***n*****/*****N*** **(%)**	***n*****/*****N*** **(%)**
	20/66 (30.3)	14/79 (17.7)

*EHF, extended high frequencies*.

There was a significant difference between the prevalence of hearing loss amongst the two groups (*P* = 0.000, χ2 = 14.00). Among hypertensives, 60.0% had bilateral hearing loss, whereas 53.9% of the non-hypertensive control group had bilateral hearing loss. To ascertain the degree of hearing loss, ear-specific degree of hearing loss was reported (Table 3). The majority of the hypertensives had mild (19.8%) to moderate (6.1%) hearing loss. Whilst the majority of the non-hypertensive participants had mild hearing loss (7.5%). The pure tone average for the extended-high frequencies (PTA_9,10,11.2,12.5,14,16_) was calculated using both participants' ears. The PTA_9,10,11.2,12.5,14,16_ among the hypertensive group was 44.1 ± 19.2 dB HL, and 20.0 ± 18.3 dB HL among the control group.

A higher prevalence of tinnitus (41.5%) was found in the hypertensive group as compared to the non-hypertensive control group (22.8%) (Table 3). The results showed a significant difference in the prevalence of tinnitus between the two groups (*P* = 0.008, χ2 = 7.09). The majority of the hypertensive participants with tinnitus reported bilateral tinnitus (54.6%), whilst the majority of non-hypertensives with tinnitus reported unilateral tinnitus (52.4%). A hissing tinnitus sound was the most prevalent tinnitus sound amongst the hypertensive (43.2%) and non-hypertensive control participants (33.3%). The proportion of tinnitus cases among participants without hearing loss was determined in both groups. In this study, 20 out of 66 (30.3%) hypertensive adults had tinnitus without hearing loss compared to 14 out of 79 (17.7%) non-hypertensive adults (Table 3).

### Factors Associated With Auditory Deficits Among Hypertensives

The odds ratios of auditory deficits—hearing loss and tinnitus—in adults with hypertension are shown in Tables 4, 5, respectively. The results from the multivariate regression analysis in Table 4 showed that after adjusting for other variables the odds of hearing loss in hypertensive adults who are between 50 and 55 years old were 3.35 higher than those who were between 18 and 29 years of age (AOR = 3.35; 95% CI: 1.32–8.50; *P* = 0.011). Having grade 2 hypertension increased the odds of having hearing loss by 4.18 (AOR = 4.18; 95% CI: 1.02–17.10; *P* = 0.048). The odds of hearing loss in hypertensive adults who were on antihypertensive medication were 3.18 higher than those not on hypertensive medication (AOR = 3.18; 95% CI: 1.02–9.87; *P* = 0.045).

**Table 4:** Univariate and multivariate logistic regression analysis of factors associated with hearing loss among hypertensives.

**Variable**	**Hearing loss^a^ *n*/*N* (%)**	**Univariable analysis**			**Multivariable analysis^b^**		
		**OR**	**95% CI**	***P*-value**	**aOR**	**95% CI**	***P*-value**
**Sex**
Male	13/48 (27.1)	1.0					
Female	27/58 (46.6)	2.34	1.03–5.32	**0.042**			
**Age (years)**
18–29	1/8 (12.5)	1.0					
30–39	5/22 (22.7)	2.06	0.20–20.96	0.542			
40–49	9/32 (28.1)	2.74	0.29–25.54	0.376			
50–55	25/44 (56.8)	9.21	1.04–81.36	**0.046**	3.35	1.32–8.50	**0.011**
**Hypertension duration**
0–5 years	14/47 (29.8)	1.0					
6–10 years	8/27 (29.6)	0.99	0.35–2.80	0.989			
>10 years	18/32 (56.3)	3.03	1.19–7.74	**0.020**	1.95	0.74–5.18	0.177
**Hypertension grade**
Controlled (SPB <140/DBP <90)	9/51 (17.6)	1.0					
Grade 1 (SPB ≥ 140–159/DBP ≥ 90–99)	15/30 (50.0)	4.67	1.69–12.88	**0.003**			
Grade 2 (SPB ≥ 160–179/DBP ≥ 100–109)	8/12 (66.7)	9.33	2.30–37.83	**0.002**	4.18	1.02–17.10	**0.048**
Grade 3 (SPB ≥ 180/DBP ≥ 110)	8/13 (61.5)	7.47	1.98–28.21	**0.003**			
**Antihypertensive medication**
No	5/28 (17.9)	1.0					
Yes	35/78 (44.9)	3.74	1.29–10.86	**0.015**	3.18	1.02–9.87	**0.045**

a *Hearing loss defined as four-frequency pure tone average of ≥20 dB HL in at least one ear*.

b *A manual forward selection stepwise was used to shortlist all predictor variables with a significance level of p < 0.25. A p-value of <0.05 was used as the inclusion criterion for the different factors into the multivariable model. P-values that are < 0.05 are marked in bold*.

**Table 5:** Univariate and multivariate logistic regression analysis of factors associated with tinnitus among hypertensives.

**Variable**	**Tinnitus *n*/*N* (%)**	**Univariable analysis**			**Multivariable analysis^a^**		
		**OR**	**95% CI**	***P*-value**	**aOR**	**95% CI**	***P*-value**
**Sex**
Male	16/48 (33.3)	1.0					
Female	27/58 (46.6)	1.74	0.79–3.84	0.169			
**Age (years)**
18–29	2/8 (25.0)	1.0					
30–39	8/22 (36.4)	1.71	0.28–10.59	0.562			
40–49	13/32 (40.6)	2.05	0.36–11.80	0.420			
50–55	20/44 (45.5)	2.50	0.45–13.78	0.293			
**Hypertension duration**
0–5 years	20/47 (42.6)	1.0					
6–10 years	9/27 (33.3)	0.68	0.25–1.81	0.435			
>10 years	14/32 (43.8)	1.05	0.42–2.60	0.916			
**Hypertension grade**
Controlled (SPB <140/DBP <90)	10/51 (19.6)	1.0					
Grade 1 (SPB ≥ 140–159/DBP ≥ 90–99)	17/30 (56.7)	5.36	1.97–14.57	**0.001**			
Grade 2 (SPB ≥ 160–179/DBP ≥ 100–109)	7/12 (58.3)	5.74	1.50–21.92	**0.011**			
Grade 3 (SPB ≥ 180/DBP ≥ 110)	9/13 (69.3)	9.23	2.35–36.15	**0.001**	3.90	1.12–12.64	**0.033**
**Antihypertensive medication**
No	10/28 (35.7)	1.0					
Yes	33/78 (42.3)	1.32	0.54–3.23	0.543			

a *A manual forward selection stepwise was used to shortlist all predictor variables with a significance level of p < 0.25. A p-value of <0.05 was used as the inclusion criterion for the different factors into the multivariable model. P-values that are <0.05 are marked in bold*.

Table 5 shows the multivariate model assessing the relationship between hypertensive characteristics and tinnitus status among hypertensives. After adjusting for other variables, the odds of tinnitus in adults who had grade 3 hypertension were 3.90 higher than those with controlled hypertension (AOR = 3.90; 95% CI: 1.12–12.64; *P* = 0.033).

## Discussion

This study aimed at examining the association between hypertension and auditory deficits (hearing loss and tinnitus) by estimating the prevalence of auditory deficits among hypertensive adults attending outpatient clinics in a tertiary hospital in South Africa. The prevalence of auditory deficits in hypertensive adults was significantly higher than in the non-hypertensive control group. This study found a hearing loss prevalence of 37.4% among hypertensive adults. Factors associated with hearing loss in this group included being between 50 and 55 years of age, having grade 2 hypertension, and being on antihypertensive medication.

This study's prevalence of hearing loss among the hypertensive group was in line with previously reported prevalence of 46.8% (35), 36.7% (13), 38.5% (16), and 12.83% (19). The current study defined hearing loss as per the recently revised WHO hearing loss classification system (27). This classification system is the adoption of the earlier WHO system, and differs from the earlier system in that measurement of onset of hearing loss was lowered from 26 to 20 dB. The revised grades of hearing loss and their corresponding hearing thresholds have been described in detail in the materials and methods section of this study. Although previous similar studies utilized the earlier WHO's grades of hearing impairment to classify normal hearing as a four-frequency pure-tone average (500, 1,000, 2,000, and 4,000 Hz) of ≤25 dB HL (36), the prevalence of hearing loss in their studies are similar to that found in the current study. The decision to lower the upper limit of normal hearing from 26 to 20 dB was informed by extensive clinical experience and evidence in literature demonstrating the negative impacts of hearing loss above 20 dB HL such as difficulty hearing conversational speech in noise (27, 36). Unaddressed hearing loss—of any degree—among hypertensive adults has the potential to greatly impact their quality of life, their ability to communicate without difficulty, and in turn their ability to obtain and retain employment. As stated in the International Classification of Functioning, Disability and Health (ICF), an individual with the smallest reduction in hearing sensitivity has a potentially “disabling” condition (27).

In this study, the most common degree of hearing loss was mild hearing loss. This is in agreement with previous findings from other studies which reported mild hearing loss as the most common hearing loss degree (13, 16). Both Yikawe et al. and Agarwal et al. categorized mild hearing loss as per the earlier WHO classification of hearing loss (26–40 dB) (13, 16). Nonetheless, a closer inspection of the hypertensive group's audiometric results from Yikawe et al. and Agarwal et al. (Figure 1 in Yikawe et al., and Table 3 in Agarwal et al., respectively) still indicates a mild degree of hearing loss when the revised WHO classification of hearing loss is applied (27). Thus, it can be argued that mild hearing loss is the most common degree of hearing loss among hypertensives. In accordance to previous studies, the current study excluded participants with an air-bone gap of ≥10 dB HL (13, 16, 19). As a result, all participants who presented with hearing loss in this study had sensorineural hearing loss.

With respect to predictors for hearing loss in the hypertensive group, the current study found that being between ages 50–55 years increased the odds of having hearing loss by more than 3-folds (AOR = 3.35; 95% CI: 1.32–8.50; *P* = 0.011). Additionally, age is an uncontested risk factor for hearing loss. An ongoing cross-sectional survey with audiometric testing in the United States of America found that the odds of hearing loss were 33-fold higher (95% CI: 10–112) in the 50–59 years of age population compared to 20–29 years age group (37). Our study matched the sex and age of the hypertensive and non-hypertensive participants—thus, age-related hearing loss may not be used to explain the hearing loss among the hypertensive group in the current study. Our study also found that hypertensive participants with grade 2 hypertension had increased odds of hearing loss (AOR = 4.18; 95% CI: 1.02–17.10; *P* = 0.048). Higher hypertension grades have been shown to exacerbate hearing loss in previous findings (13). The current study also found that odds of hearing loss were higher in hypertensive adults on antihypertensive medication (AOR = 3.18; 95% CI: 1.02–9.87; *P* = 0.045). The effects of antihypertensive medication on hearing are varied. In small studies conducted over four decades ago, furosemide - a loop diuretic, has been associated with reversible sudden sensorineural hearing loss that can be permanent in some cases (38–40). On the contrary, a recent prospective cohort study of 54,721 women did not find an association between use of thiazide diuretics and furosemide with increased risk of hearing loss (41). Similarly, a recent hospital-based cross-sectional study of 250 hypertensive participants and 250 non-hypertensive controls aged 20–59 years, did not find a significant association between class of antihypertensives used by the hypertensive participants and hearing loss (42). Our cross-sectional study only reported on whether hypertensive participants were on antihypertensives and not the specific medication they were on nor the duration of treatment. This was a limitation to our study; thus, it is important for future studies to investigate this association further.

There is a dearth of research employing extended-high frequency (EHF) audiometry in evaluating the link between hypertension and hearing loss. The current study made use of EHF audiometry to compare EHF hearing sensitivity of both the hypertensive and non-hypertensive control group. Our study found more than 20 dB worse PTA_9,10,11.2,12.5,14,16_ hearing sensitivity between the hypertensive and non-hypertensive group (44.1 vs. 20.0 dB). Similar findings were reported by Baiduc et al. with worse EHF hearing sensitivity in the hypertensive group compared to the non-hypertensive group (18.86 vs. 14.02 dB) (43). These results may suggest the possibility of early-onset of hypertension-related hearing loss in the extended-high frequencies (EHF) that could be monitored and detected early using EHF audiometry.

The current study employed diagnostic pure tone audiometry in the conventional frequencies (250–8,000 Hz) and in the EHF in order to assess the participants' hearing capacity. Pure tone audiometry is considered the “gold standard” of hearing function assessment and has been recommended for epidemiological use (27). However, it has been demonstrated in previous studies that tinnitus patients with normal audiograms could present with “hidden hearing loss”; and “hidden hearing loss” can be evaluated by measuring auditory brainstem responses (ABR) (44). Therefore, there is a need for further studies to investigate the association between hypertension and hearing loss using objective audiological tests such as ABR in order to ascertain the prevalence of hearing loss amongst hypertensive adults.

With respect to tinnitus, the current study found a tinnitus prevalence of 41.5% among hypertensive adults. Grade 3 hypertension was associated with tinnitus. The prevalence of tinnitus in this study was slightly lower than that of Mondelli and Lopes with a reported tinnitus prevalence of 43.7% among hypertensives (23). Although the cause-and-effect relationship is uncertain, there is extensive literature demonstrating an association between hypertension and tinnitus (22, 45). However, in most studies showing an association between tinnitus and hypertension, hearing loss is a variable. According to Lee et al., 85–96% of individuals with tinnitus have some degree of hearing impairment (24). Thus, to ascertain the association between hypertension and tinnitus, the proportion of hypertensives with tinnitus without hearing loss was determined. In the current study 30.3% of hypertensive adults had tinnitus without hearing loss compared to 17.7% non-hypertensive adults. Our study highlighted that grade 3 hypertension is a predictor variable for tinnitus among hypertensive adults. The authors could not identify published studies exploring the association between hypertension characteristics and tinnitus. Grade 3 hypertension as a predictor variable for tinnitus is biologically plausible due to hypertension's effect as a high output state (46).

The current research was the first non-multifactorial study dedicated to the investigation of the association between hypertension and auditory deficits in South Africa. Our study has a number of strengths that include the use of extended-high frequency audiometric measurements and the revised WHO hearing loss classification system with a strict upper limit (normal hearing defined as <20 dB HL). As such, the prevalence of hearing loss among hypertensive adults could not be underestimated. Another strength of the current study was the utilization of retrospective medical file review in order to obtain participants' medical history—this decreased the likelihood of recall bias. Lastly, to the authors knowledge, this was the first study to determine the proportion of tinnitus among hypertensive adults without hearing loss. This ensured the determination of an unconfounded association between hypertension and tinnitus. The current study had some limitations. Firstly, the study population was predominately black Africans; further investigations are required to evaluate this association in other population groups. Secondly, our study employed a cross-sectional study design collecting data at one point in time, as a result the cause-effect relationship could not be inferred. Lastly, the duration of hypertension may have been underestimated due to the asymptomatic nature of hypertension. Thus, some participants may have had hypertension longer than their date of diagnosis.

## Conclusion

Auditory deficits can be disabling; thus, the identification of potential modifiable risk factors is of public health importance. The current study found a 37.4% prevalence of hearing loss, and a 41.5% prevalence of tinnitus among hypertensive adults attending a tertiary hospital in South Africa. The prevalence of tinnitus among hypertensive adults without hearing loss was found to be 30.3%. The most common characteristic of the hearing loss was a bilateral mild sensorineural hearing loss with decreased extended-high frequency hearing sensitivity. Being between 50 and 55 years of age, having grade 2 hypertension, and being on antihypertensive medication were factors associated with increased odds of hearing loss. Furthermore, grade 3 hypertension was associated with increased tinnitus prevalence. Findings suggest an association between hypertension and auditory deficits, demonstrating a need for the integration of hearing healthcare services for hypertension management. The monitoring of hypertensive adults' hearing status using EHF audiometry offers the possibility of early hearing loss detection and intervention in order to prevent hearing loss in the conventional frequencies (250–8,000 Hz) or introduce timely rehabilitation.

## Data Availability Statement

The raw data supporting the conclusions of this article will be made available by the authors, without undue reservation.

## Ethics Statement

The studies involving human participants were reviewed and approved by University of Pretoria Health Sciences Research Ethics Committee (Ethics Reference No: 287/2021). The patients/participants provided their written informed consent to participate in this study.

## Author Contributions

HR collected the data for the study and analyzed the data. HR and SP contributed to the final write up of the manuscript. All authors contributed to the idealization of the study, the protocol, and the study design. All authors contributed to the article and approved the submitted version.

## Conflict of Interest

The authors declare that the research was conducted in the absence of any commercial or financial relationships that could be construed as a potential conflict of interest.

## Publisher's Note

All claims expressed in this article are solely those of the authors and do not necessarily represent those of their affiliated organizations, or those of the publisher, the editors and the reviewers. Any product that may be evaluated in this article, or claim that may be made by its manufacturer, is not guaranteed or endorsed by the publisher.

## Abbreviations

ACCF/AHA: American College of Cardiology Foundation/American Heart Association Task Force on Practice Guidelines
DBP: Diastolic blood pressure
EHF: Extended high frequency
EQ-5D: EuroQol-5 Dimension
HBP: High blood pressure
HICs: High-income countries
HRQoL: Health-related quality of life
LMICs: Low- and middle-income countries
PTA: Pure tone average
SBP: Systolic blood pressure
TOD: Target organ damage
USA: United States of America
WHO: World Health Organization.
